# The Impact of NAFLD and Waist Circumference Changes on Diabetes Development in Prediabetes Subjects

**DOI:** 10.1038/s41598-019-53947-z

**Published:** 2019-11-21

**Authors:** Jiwoo Lee, Yun Kyung Cho, Yu Mi Kang, Hwi Seung Kim, Chang Hee Jung, Hong-Kyu Kim, Joong-Yeol Park, Woo Je Lee

**Affiliations:** 1 Department of Internal Medicine, Asan Medical Center, University of Ulsan College of Medicine, Seoul, Republic of Korea; 2 Department of Health Screening and Promotion Center, Asan Medical Center, University of Ulsan College of Medicine, Seoul, Republic of Korea

**Keywords:** Endocrinology, Medical research, Risk factors

## Abstract

The aim of this study was to investigate the association of nonalcoholic fatty liver disease (NAFLD) with diabetes and the impact of waist circumference (WC) changes in subjects with prediabetes. We enrolled 6240 subjects with prediabetes who underwent health check-ups in 2007 and revisited our hospital at least once for a follow-up examination between 2008 and 2013. Subjects were stratified by WC changes into three groups. The relative risks (RRs) for diabetes according to the NAFLD status and WC change were evaluated. The prevalence of NAFLD was 45.4% (2830/6240). During follow-up, the incidence of diabetes was 8.1% (505/6240). Subjects with NAFLD had a higher incidence of diabetes and the adjusted RRs were 1.81 (95% confidence interval [CI], 1.47 to 2.21), after adjustment for potential confounding factors. The adjusted RRs were related to WC changes. The adjusted RRs for diabetes according to tertiles of WC change (first, second, and third tertile) were 1.64 (95% CI, 1.08 to 2.49), 1.73 (95% CI, 1.28 to 2.34), and 2.04 (95% CI, 1.42 to 2.93), respectively. NAFLD has significantly increased risk of incident diabetes in subjects with prediabetes. The risk for diabetes is gradually increased with tertiles of WC change.

## Introduction

Nonalcoholic fatty liver disease (NAFLD) has become the most common type of liver disease worldwide^1^. In Asian countries, including Korea, the prevalence of NAFLD is growing gradually but steadily along with increases in aging, obesity, and a more westernized lifestyle^2^. NAFLD is not only associated with liver-related diseases, but also well-known independent risk factors for diabetes, and has the potential therefore to cause multiple clinical complications and significant cost burdens^3–5^.

Prediabetes predisposes the affected individual to a high risk of progression to diabetes. Indeed, each year, 5–10% of patients with prediabetes progress to diabetes^6^ and eventually almost 70% of prediabetes cases will develop the full condition, according to the American Diabetes Association (ADA) expert panel^7^. The progression from prediabetes to diabetes is of great interest as a primary prevention strategy, based on epidemiological and clinical evidence.

NAFLD also coexists in a substantial percentage of patients with prediabetes^8^ and has an additive effect on the future diabetes risk in patients with impaired fasting glucose (IFG)^3^. Few studies have been performed however on the risk of diabetes in subjects with prediabetes who also have NAFLD.

Obesity, particularly abdominal obesity, is one of the conditions that is frequently associated with NAFLD. The waist circumference (WC) is an anthropometric measurement of obesity and can easily be assessed in a health check-up and in other clinical settings. The WC is related to abdominal (visceral) adiposity, which is considered to be an important predictor of development diabetes and NAFLD^9,10^.

Notably, most previous studies of the relationship between WC and diabetes have been cross-sectional, and have reported on the effects of WC on diabetes onset based on a cutoff value^11^. Hence, the mechanisms by which WC changes affect the development of diabetes are still elusive.

In summary, few studies to date have been performed in prediabetics with NAFLD to evaluate the impacts on the risk of diabetes. In addition, the effects of long-term WC changes on the development of diabetes in prediabetes cases with NAFLD have not been established. In view of this, we aimed in our current study to more fully investigate the association between NAFLD and diabetes, and also determine the effects of WC changes in subjects with prediabetes.

## Results

The baseline characteristics of the 6240 study subjects according to their NAFLD status are presented in Table 1. The mean age of this cohort was 50.71 ± 8.18 years and most of the subjects were male (73.6%). The mean follow-up duration was 4.30 ± 1.91 years and 93.3% (5826/6240) of patients were followed up for more than 2 years. The mean BMI was 24.60 ± 2.78 kg/m^2^, the mean WC was 84.02 ± 8.22 cm, and the percentage of overweight/obesity was 72.0%.

**Table 1:** Baseline Characteristics of the Study Subjects According to their NAFLD Status.

Characteristics	NAFLD		*P*
	Yes (n = 2830)	No (n = 3410)	
Age (years)	50.49 ± 8.13	50.90 ± 8.22	0.049
Height (cm)	168.46 ± 7.75	166.08 ± 8.15	<0.001
Weight (kg)	73.62 ± 10.21	65.18 ± 9.96	<0.001
BMI (kg/m2	25.87 ± 2.57	23.54 ± 2.50	<0.001
HbA1c (%)	5.66 ± 0.36	5.58 ± 0.36	<0.001
FPG (mg/dL)	103.72 ± 8.75	101.43 ± 8.50	<0.001
Waist circumference (cm)	88.08 ± 6.96	80.66 ± 7.65	<0.001
SBP (mmHg)	123.22 ± 13.61	119.40 ± 14.11	<0.001
DBP (mmHg)	77.09 ± 8.80	74.21 ± 8.75	<0.001
Total cholesterol (mg/dL)	200.91 ± 33.63	192.74 ± 32.39	<0.001
Triglycerides (mg/dL)	176.92 ± 108.29	118.96 ± 63.81	<0.001
HDL cholesterol (mg/dL)	50.94 ± 11.31	58.68 ± 14.36	<0.001
LDL cholesterol (mg/dL)	131.60 ± 29.85	123.10 ± 28.63	<0.001
Insulin (µIU)^†^	10.11 ± 5.14	6.86 ± 3.41	<0.001
HOMA-IR^†^	2.60 ± 1.38	1.73 ± 0.90	<0.001
AST (IU/L)	27.41 ± 19.86	23.30 ± 10.43	<0.001
ALT, (IU/L)	31.62 ± 32.22	20.96 ± 16.60	<0.001
Creatinine (mg/dL)	0.93 ± 0.16	0.89 ± 0.17	<0.001
%			
Sex			<0.001
Male	2351 (83.1)	2243 (65.8)	
Female	479 (16.9)	1167 (34.2)	
Current smoker			<0.001
Yes	1916 (67.7)	1777 (52.1)	
No	914 (32.3)	1633 (47.9)	
Current alcohol drinker			<0.001
Yes	1580 (55.8)	1618 (47.4)	
No	1250 (44.2)	1792 (52.6)	
Overweight/obese			<0.001
Yes	2518 (89.0)	1975 (57.9)	
No	312 (11.0)	1435 (42.1)	
Hypertension			<0.001
Yes	1302 (46.0)	1107 (32.5)	
No	1528 (54.0)	2303 (67.5)	

BMI, body mass index; SBP, systolic blood pressure; DBP, diastolic blood pressure; FPG, fasting plasma glucose; HbA1c, hemoglobin A1c; LDL cholesterol, low-density lipoprotein cholesterol; HDL cholesterol, high-density lipoprotein cholesterol; HOMA-IR, Homeostatic Model Assessment for Insulin Resistance; AST, aspartate aminotransferase; ALT, alanine aminotransferase.

^†^Insulin levels and HOMA-IR were not available for 1375 patients.

The prevalence of NAFLD was 45.4% (2830/6240). The mean age was higher in the subgroup of patients without NAFLD (P = 0.049). A comparison of the parameters among the subgroups showed that the NAFLD group had a higher BMI, WC, glycemic levels (FPG and HbA1c), lipid level, liver enzyme level, and blood pressure (P < 0.001). The proportion of overweight/obesity was also significantly higher in the NAFLD group. Moreover, the NAFLD subjects had a higher prevalence of current smoker, current alcohol drinkers, and cases of hypertension (P < 0.001).

### Comparison of the baseline characteristics according to outcome

The subjects with prediabetes were categorized into two subgroups according to the development of diabetes. The baseline characteristics of these subjects by subgroup are listed in Table 2. The subjects who developed diabetes were older and demonstrated a higher BMI, WC, FPG, HbA1c, triglycerides, AST, and ALT and a lower HDL cholesterol level (P < 0.001). In addition, the diabetes cases were significantly male dominant and had a higher percentage of current smokers, and of subjects with overweight/obesity, hypertension, and in particular a higher percentage of NAFLD (P < 0.001). The blood pressure, total cholesterol, LDL-cholesterol, and creatinine levels did not differ among these two subgroups, nor did the percentage of current alcohol drinkers.

**Table 2:** Baseline Characteristics of the Study Subjects According to Incident Diabetes.

Characteristics	Diabetes (n = 505)	No Diabetes (n = 5735)	*P*
Age (year)	52.08 ± 8.17	50.59 ± 8.17	<0.001
Height (cm)	167.70 ± 7.61	167.11 ± 8.09	0.114
Weight (kg)	72.23 ± 10.78	68.73 ± 10.88	<0.001
BMI (kg/m^2^	25.60 ± 2.86	24.51 ± 2.76	<0.001
HbA1c (%)	5.94 ± 0.32	5.59 ± 0.35	<0.001
FPG (mg/dL)	109.69 ± 10.32	101.83 ± 8.23	<0.001
Waist circumference (cm)	87.27 ± 7.87	83.74 ± 8.19	<0.001
SBP (mmHg)	122.24 ± 14.12	121.04 ± 14.01	0.064
DBP (mmHg)	75.82 ± 8.92	75.49 ± 8.89	0.422
Total cholesterol (mg/dL)	197.35 ± 35.13	196.36 ± 33.04	0.521
Triglycerides (mg/dL)	173.54 ± 108.95	142.75 ± 89.40	<0.001
HDL cholesterol (mg/dL)	51.05 ± 11.42	55.53 ± 13.74	<0.001
LDL cholesterol (mg/dL)	128.21 ± 31.06	126.85 ± 29.35	0.320
Insulin (µIU)†	9.09 ± 4.81	8.26 ± 4.55	0.001
HOMA-IR†	2.48 ± 1.37	2.09 ± 1.20	<0.001
AST (IU/L)	27.80 ± 14.78	24.93 ± 15.62	<0.001
ALT (IU/L)	31.21 ± 23.38	25.32 ± 25.61	<0.001
Creatinine (mg/dL)	0.92 ± 0.17	0.91 ± 0.17	0.052
%			
Sex			<0.001
Male	406 (80.4)	4188 (73.0)	
Female	99 (19.6)	1547 (27.0)	
Current smoker			<0.001
Yes	340 (67.3)	3353 (58.5)	
No	165 (32.7)	2382 (41.5)	
Current alcohol drinker			0.912
Yes	260 (51.5)	2938 (51.2)	
No	245 (48.5)	2797 (48.8)	
Overweight/obesesity			<0.001
Yes	424 (84.0)	4069 (71.0)	
No	81 (16.0)	1666 (29.0)	
Hypertension			<0.001
Yes	234 (46.3)	2175 (37.9)	
No	271 (53.7)	3560 (62.1)	
NAFLD			<0.001
Yes	344 (68.1)	2486 (43.3)	
No	161 (31.9)	3249 (56.7)	

BMI, body mass index; SBP, systolic blood pressure; DBP, diastolic blood pressure; FPG, fasting plasma glucose; HbA1c, hemoglobin A1c; LDL cholesterol, low-density lipoprotein cholesterol; HDL cholesterol, high-density lipoprotein cholesterol; HOMA-IR, Homeostatic Model Assessment for Insulin Resistance; AST, aspartate aminotransferase; ALT, alanine aminotransferase; NAFLD, nonalcoholic fatty liver disease.

^†^Insulin and HOMA-IR levels were not available for 1375 patients.

### Incidence of diabetes according to the NAFLD status

**Figure 1:**
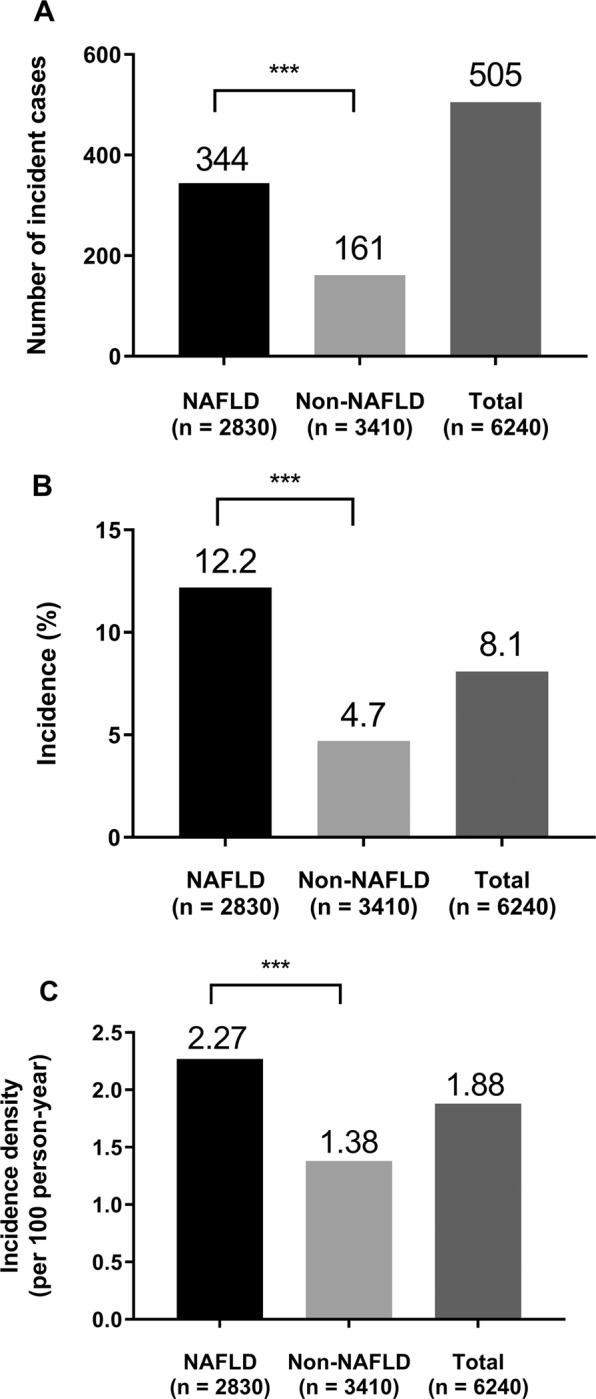
Incidence of Diabetes According to the NAFLD status. (**A**) Number of incident cases. (**B**) Incidence (%). (**C**) Incidence density (per 100 person-years). ***P < 0.001.

Subjects with NAFLD at baseline had a higher incidence of diabetes; 12.16% (344/505) for the positive cases compared to 4.72% for the negative cases (161/3410) (Fig. 1A & B). The total incidence of diabetes was therefore 8.09% (505/6240). Additionally, the incidence density was 2.27 per 100 person-years in subjects with NAFLD and 1.38 per 100 person-years in subjects without NAFLD. The total incidence density was 1.88 per 100 person-years for diabetes (Fig. 1C).

### Relative risks for diabetes onset according to the NAFLD status and waist circumference changes

Multivariate models for the development of diabetes in the study subjects with prediabetes (Table 3) revealed that the presence of NAFLD was a significant independent risk factor. The prediabetes subjects with NAFLD had a significantly increased RR for diabetes incidence, which was 2.76 (95% CI, 2.29 to 3.33) in the unadjusted model. Similarly, the RR was 2.66 (95% CI, 2.20 to 3.22) after adjustments for lifestyle-related factors. Finally, these significantly increased RRs were sustained by 1.81 (95% CI, 1.47 to 2.21) after adjustment for age, sex, smoking, alcohol drinking status, HbA1c, ALT, TG, HDL-C, BMI, and systolic BP at baseline.

**Table 3:** RRs of Diabetes According to NAFLD and WC Changes in the Subjects with Prediabetes (Tertile Stratification).

	Model 1		Model 2		Model 3		Model 4	
	RR (95% CI)	*P*	RR (95% CI)	*P*	RR (95% CI)	*P*	RR (95% CI)	*P*
Total population	2.76 (2.29–3.33)	<0.001	2.66 (2.20–3.22)	<0.001	2.29 (1.88–2.79)	<0.001	1.81 (1.47–2.21)	<0.001
**WC change (cm)**								
1^st^ (<0.0)	2.36 (1.61–3.46)	<0.001	2.34 (1.59–3.44)	<0.001	2.06 (1.61–2.91)	<0.001	1.64 (1.08–2.49)	0.020
2^nd^ (0.0 to 3.5)	2.76 (1.97–3.87)	<0.001	2.65 (1.88–3.74)	<0.001	2.17 (1.43–3.29)	<0.001	1.73 (1.28–2.34)	<0.001
3^rd^ (>3.5)	2.95 (2.23–3.90)	<0.001	2.86 (2.15–3.79)	<0.001	2.37 (1.66–3.38)	<0.001	2.04 (1.42–2.93)	<0.001

RR, relative risk; CI, confidence interval; WC, waist circumference.

Model 1: no adjustment.

Model 2: adjusted for age, sex, smoking, and alcohol drinking at baseline.

Model 3: adjusted for the variables in model 2 plus ALT, TG, HDL-C, BMI, and systolic BP at baseline.

Model 4: adjusted for the variables in model 3 plus HbA1c at baseline.

All of the subjects were classified into three groups according to the WC change, i.e. first tertile (n = 2117, median: −2.0 cm, range: −18.0 to −0.4 cm), second tertile (n = 2066, median: 2.0 cm, range: 0.0 to 3.5 cm), and third tertile (n = 2055, median: 6 cm, range: 3.6 to 21.0 cm). The RRs of diabetes were found to be significantly associated with these WC changes such that the RRs gradually increased over the follow-up period as follows: first tertile (RR = 2.36, 95% CI = 1.61–3.46), second tertile (RR = 2.76, 95% CI = 1.97–3.87), and third tertile (RR = 2.95, 95% CI = 2.23–3.90) (*P* for trend<0.01). After multivariate adjustment, similar results were observed. The adjusted RRs for diabetes according to the three WC change groups were 1.64 (95% CI, 1.08 to 2.49), 1.73 (95% CI, 1.28 to 2.34), and 2.04 (95% CI, 1.42 to 2.93), respectively (*P* for trend <0.01). In addition, when WC changes were divided into three groups, i.e. <−1.0 cm, −1.0 to 1.0 cm, and >1.0 cm, the results were similar (Supplementary Table 1).

Moreover, we analyzed the RR of diabetes according to NAFLD and WC changes stratified by sex (Supplementary Table 2). For both men and women, NAFLD still had significantly increased RRs for diabetes incidence. In men, the RR of NAFLD for diabetes according to the three WC change groups showed a gradual increase depending on WC change, after adjusting for potential confounding factors (*P* for trend 0.05). In women, the highest tertiles of WC change were significantly associated with risk of diabetes in prediabetes with NAFLD.

We also conducted sensitivity analysis by excluding patients followed for less than 2 years and the results were similar. The RR for diabetes was 3.02 (95% CI, 2.46 to 3.72) in the unadjusted model and 2.02 (95% CI, 1.62 to 2.52) in the adjusted model. When WC changes were divided into three groups, the trend of increased RR was consistent (Supplementary Table 4).

## Discussion

We have found in our present study that the presence of NAFLD at baseline is associated with a higher risk of diabetes in subjects with prediabetes. Interestingly, WC increases were also found to be related to the risk of developing diabetes in subjects with prediabetes and NAFLD.

Because of less healthy and more westernized lifestyles, the prevalence of NAFLD and diabetes has increased over the past decade in a number of regions including Asia^2,12^, resulting in increased health problems for those countries^13^. In a recent meta-analysis, the overall prevalence of NAFLD in Asia was 28.5%^14^. A cohort study in Korea reported a 27% prevalence of NAFLD in Korea^15^. When it comes to the prevalence of NAFLD in prediabetes state, a Japanese study reported the prevalence of NAFLD in subjects with impaired fasting glucose as 43%^8^. In a previous study in Korea, the prevalence of NAFLD in prediabetes subjects was reported as 47%^16^. In this study, the prevalence of NAFLD in prediabetes subjects was 45.4% which is similar to previous results.

NAFLD has been considered an important predictor of incident diabetes, although the RR or hazard ratio (HR) values have varied across studies^3,5,17^. A longitudinal study in Korea has reported a HR of NAFLD for diabetes of 1.33 (95% CI, 1.1 to 1.7)^3^. A prior study from Japan has also reported that NAFLD is related to the incidence of diabetes, with an OR of 2.37 (95% CI, 1.60 to 3.52)^17^. A previous meta-analysis summarizing 19 observational studies has additionally demonstrated a significant relationship between NAFLD and the risk of incident diabetes, with an overall HR of 2.22 (95% CI 1.84 to 2.60)^5^.

It is notable however that most of the previously published studies showing an association between NAFLD and the risk of incident diabetes included subjects with both a normal glucose level and prediabetes at baseline^5^. To the best of our knowledge, only one study to date has reported an association between NAFLD and impaired fasting glucose on the development of diabetes^3^. Consistent with previous results, our present findings have indicated a positive association between NAFLD and diabetes onset (RR, 1.81; 95% CI, 1.47 to 2.21). Our current analyses have thus filled the previous gaps in the relationship between NAFLD and diabetes among subjects with prediabetes, showing that, even in prediabetes patients with NAFLD, there is still a significant association between NAFLD and diabetes.

We also demonstrated the impact of WC changes on the association between NAFLD and diabetes in our present subjects with prediabetes. We found from our analysis that the RRs for diabetes were positively associated with WC changes. Subjects in the lowest tertile of WC change had a lower RR, and those in the highest tertile of WC change during follow-up had a higher RR for diabetes. Previous studies have reported WC changes can affect the incidence of metabolic diseases including diabetes^18–22^. A prior 9-year follow-up report, the Data from an Epidemiological Study on the Insulin Resistance Syndrome (DESIR) study, demonstrated that cardiometabolic factors were poorer in subjects with an increased WC^18^. Another study of this same cohort reported that an increased WC is an important risk factor for the progression of type 2 diabetes in individuals with prediabetes^19^. Studies from the United States, Japan, and China also reported that changes in WC were related to the subsequent risk of diabetes^20–22^. Our current findings are consistent with these previous studies.

To the best of our knowledge, our present report is the first to investigate the impact of WC changes on the association between NAFLD and incident diabetes in subjects with prediabetes. Whether an increased WC aggravated the fatty liver or affected both NAFLD and diabetes was difficult to clarify, however. An increased WC represents visceral fat accumulation that contributes to hepatic inflammation and oxidative damage, which is known to aggravate hepatic steatosis^23^. In addition, visceral fat excess has been found to be related to decreased insulin sensitivity and result in poor glycemic control, thereby associated with the development of diabetes^24^. Although we could not clearly discriminate the influence of WC changes on NAFLD and/or diabetes in our current study series, we observed that our NAFLD and prediabetes subjects who had a decreased WC had a lower RR, and those with an increased WC had a higher RR, for diabetes onset. Hence, efforts to decrease the WC in subjects with NAFLD and prediabetes might delay or prevent the development of diabetes.

There were several limitations of our present study of note. First, our subjects were voluntarily recruited during general health check-ups and are not representative of the general population. Second, because the frequency of follow-up visits was not predetermined, the timing of the onset of diabetes in our cohort could be inaccurate. Third, the diagnosis of NAFLD was made by ultrasonography rather than biopsy. Fourth, since an alcohol consumption history could not be obtained quantitatively, it was impossible to clearly differentiate alcoholic FLD and NAFLD. Notably however, the relative contribution of alcohol consumption to the development of NAFLD is controversial^25^. Finally, we could not include the dietary habits as one of potential confounding factors in the development of diabetes. This might cause the inaccuracy of our estimated RRs for the development of diabetes.

Our study had several strengths also which are equally noteworthy. First, all physical examinations and questionnaires were carried out in accordance with a standard protocol and followed-up by trained doctors and nurses. Second, our sample size was large and the subgroup analysis thus had adequate statistical power. Furthermore, we used a longitudinal design, which helped to estimate how dynamic changes of WC may have affected the RRs for diabetes in prediabetes subjects with NAFLD. Finally, our study is the first to have evaluated the impact of WC changes on the association between NAFLD and diabetes in a large number of prediabetes cases.

In conclusion, NAFLD has significantly increased risk of incident diabetes in subjects with prediabetes. The risk for diabetes is gradually increased with tertiles of WC change. Therefore, early interventions aiming at reducing abdominal obesity are needed for patient with NAFLD and prediabetes to prevent the risk of diabetes incidence.

## Methods

### Ethics statement

In accordance with the ethical guidelines of the declaration of Helsinki and Korea Good Clinical Practice, all subjects provided written informed consent and this study was approved by the institutional review board of AMC (IRB No. 2018-0516).

### Study population

This was a retrospective cohort study comprising subjects who had undergone a general health check-up at the Health Screening and Promotion Center of Asan Medical Center (AMC, Seoul, Republic of Korea) in 2007 and revisited the clinic for a follow-up examination at least once between 2008 and 2013. Initially, 17,666 subjects were identified that satisfied these criteria. Of these subjects, 2734 were excluded because of having type 2 diabetes at the baseline examination. On the basis of the 2007 medical records, subjects were also excluded for the following reasons: absence of data (HbA1c, triglyceride, γ-glutamyl transferase (GGT), and insulin levels; n = 2573), hepatitis B (n = 569), hepatitis C (n = 72), and abnormal liver enzyme levels (n = 47). Several subjects met two or more of these exclusion criteria. Each study subject completed a questionnaire about medications, past medical or surgical history, smoking, and alcohol drinking status. Drinking status was classified by frequency per week (i.e., non-drinkers and moderate drinkers [1 or 2 times/week]), and the smoking status as noncurrent or current^26^. A final series of 6240 subjects with prediabetes were eligible for subsequent analysis.

### Clinical and laboratory measurements

We estimated clinical and laboratory data using a previously described method^26^. Briefly, physical examinations, including heights, weights, WC, and BP were measured in accordance with standard protocol. All parameters described in this study were measured in the central, certified laboratory at AMC. Blood sample data, including aspartate aminotransferase (AST), alanine aminotransferase (ALT), total cholesterol, high-density lipoprotein-cholesterol (HDL-C), low-density lipoprotein-cholesterol (LDL-C), and triglycerides (TG), were measured by Toshiba 200FR Neo analyzer (Toshiba Medical System Co., Ltd., Tokyo, Japan) using the enzymatic colorimetric method. The fasting plasma glucose (FPG) and HbA1c levels were measured by Toshiba 200 FR auto-analyzer (Tosiba) and ion-exchange high-performance liquid chromatography (Bio-Rad Laboratories, Inc., Hercules, CA), respectively, according to the manufacturer’s protocol.

### Definitions of diabetes, prediabetes and nonalcoholic fatty liver disease (NAFLD)

Subjects with diabetes were defined as those with FPG level of 126 mg/dL and/or HbA1c level of 6.5%^27^. In addition, subjects who reported the use of antidiabetic medications on a self-reporting questionnaire were regarded as diabetic^27^. Subjects with prediabetes were defined as those who met one of the following two criteria: 100 mg/dL ≤ FPG ≤ 125 mg/dL and/or 5.7% ≤ HbA1c ≤ 6.4%^27^.

Overweight and obesity were defined as BMI ≥ 23 kg/m^2^ and ≥25 kg/m^2^, respectively, according to classification of BMI in Asia by the World Health Organization^28^.

Hepatic ultrasonography was used to diagnose NAFLD (Ultrasound Systems IU22, Philips, Holland) by experts in this method. The diagnosis of a fatty liver was based on characteristic ultrasonographic findings consistent with “bright liver” and a clear contrast between hepatic and renal parenchyma, blurring of vessels, focal preservation, and stenosis of the hepatic vein lumen^9^ if the study subject had no other cause for the hepatic steatosis, such as hepatitis, alcohol, or medications.

WC changes were defined by the WC in the follow-up year minus that at baseline, and were classified as the following tertiles: <0.0 cm (first tertile), 0.0 to 3.5 cm (second tertile), or >3.5 cm (third tertile).

### Statistical analysis

Continuous variables were presented as the mean ± standard deviation (SD) and categorical variables as a percentage. Student t test were used to compare the biochemical and demographic characteristics of the subgroups stratified by their NAFLD status or outcomes for continuous variables or the χ2 test for categorical variables. Person-years were used to express the incidence of diabetes. The relative risks (RR) for diabetes in the subject with prediabetes and NAFLD were estimated by using multivariable Cox proportional hazard model. P values < 0.05 were regarded as statistically significant. All statistical analyses were performed using SPSS software version 21.0 for Windows (SPSS, Inc., Chicago, IL).

## Supplementary information

Supplementary Information File

## Data Availability

The datasets generated during and/or analyzed during the current study are available from the corresponding author on reasonable request.
