# Supplementary material for: The Impact of NAFLD and Waist Circumference Changes on Diabetes Development in Prediabetes Subjects
Source: Sci Rep. 2019 Nov 21;9:17258. doi: 10.1038/s41598-019-53947-z (PMC6872574; doi:10.1038/s41598-019-53947-z)
Supplement: Supplementary file 1 — Supplementary Information File [file 41598_2019_53947_MOESM1_ESM.docx]

**Supplementary information**

**The Impact of NAFLD and Waist Circumference Changes on Diabetes Development in Prediabetes Subjects**

Jiwoo Lee^1^, Yun Kyung Cho^1^, Yu Mi Kang^1^, Hwi Seung Kim^1^, Chang Hee Jung^1^, Hong-Kyu Kim^2^, Joong-Yeol Park^1^, Woo Je Lee^1^

^1^Department of Internal Medicine, Asan Medical Center, University of Ulsan College of Medicine, Seoul, Republic of Korea

^2^Department of Health Screening and Promotion Center, Asan Medical Center, University of Ulsan College of Medicine, Seoul, Republic of Korea

**Supplementary Table 1. RRs of Diabetes According to NAFLD and WC Changes in the Subjects with Prediabetes**

|  | **Model 1** | | **Model 2** | | **Model 3** | | **Model 4** | |
| --- | --- | --- | --- | --- | --- | --- | --- | --- |
|  | **RR (95% CI)** | ***P*** | **RR (95% CI)** | ***P*** | **RR (95% CI)** | ***P*** | **RR (95% CI)** | ***P*** |
| Total population | 2.76 (2.29–3.33) | < 0.001 | 2.66 (2.20–3.22) | < 0.001 | 2.29 (1.88–2.79) | < 0.001 | 1.81 (1.47–2.21) | < 0.001 |
| WC change (cm) |  |  |  |  |  |  |  |  |
| < –1.0 (n = 1143) | 2.24 (1.43–3.51) | < 0.001 | 2.25 (1.43–3.53) | < 0.001 | 1.97 (1.21–3.19) | 0.007 | 1.51 (0.93–2.45) | 0.096 |
| –1.0 to 1.0 (n = 1635) | 2.82 (1.97–4.03) | < 0.001 | 2.74 (1.91–3.95) | < 0.001 | 2.18 (1.48–3.23) | < 0.001 | 1.64 (1.11–2.43) | 0.013 |
| > 1.0 (n = 3460) | 2.91 (2.26–3.75) | < 0.001 | 2.76 (2.14–3.58) | < 0.001 | 2.35 (1.81–3.06) | < 0.001 | 1.99 (1.52–2.59) | < 0.001 |

RR, relative risk; CI, confidence interval; WC, waist circumference.

Model 1: no adjustment.

Model 2: adjusted for age, sex, smoking, and alcohol drinking at baseline.

Model 3: adjusted for the variables in model 2 plus ALT, TG, HDL-C, BMI, and systolic BP at baseline.

Model 4: adjusted for the variables in model 3 plus HbA1c at baseline.

**Supplementary Table 2.** **RRs of Diabetes According to NAFLD and WC Changes in the Subjects with Prediabetes, Stratified by Sex.**

|  | **Model 1** | | **Model 2** | | | **Model 3** | | **Model 4** | | |
| --- | --- | --- | --- | --- | --- | --- | --- | --- | --- | --- |
|  | **RR (95% CI)** | ***P*** | | **RR (95% CI)** | ***P*** | **RR (95% CI)** | ***P*** | | **RR (95% CI)** | ***P*** |
| Men (n = 4594) | 2.53 (2.04–3.14) | < 0.001 | | 2.55 (2.06–3.16) | < 0.001 | 2.22 (1.77–2.76) | < 0.001 | | 1.72 (1.37–2.15) | < 0.001 |
| WC change (cm) |  |  | |  |  |  |  | |  |  |
| 1^st^ (< 0.0) (n = 1363) | 2.35 (1.67–3.32) | < 0.001 | | 2.35 (1.66–3.31) | < 0.001 | 2.03 (1.37–3.01) | < 0.001 | | 1.61 (1.09–2.38) | 0.018 |
| 2^nd^ (0.0 to 3.0) (n = 1543) | 2.44 (1.60–3.73) | < 0.001 | | 2.50 (1.63–3.82) | < 0.001 | 2.05 (1.44–2.90) | < 0.001 | | 1.69 (1.18–2.41) | 0.004 |
| 3^rd^ (> 3.0) (n = 1688) | 2.83 (1.97–4.08) | < 0.001 | | 2.87 (1.99–4.15) | < 0.001 | 2.38 (1.51–3.78) | < 0.001 | | 1.83 (1.16–2.87) | 0.01 |
| Women (n = 1646) | 3.27 (2.20–4.85) | < 0.001 | | 3.12 (2.08–4.66) | < 0.001 | 2.54 (1.62–3.99) | < 0.001 | | 2.14 (1.37–3.33) | 0.001 |
| WC change (cm) |  |  | |  |  |  |  | |  |  |
| 1^st^ (< 2.0) (n = 520) | 2.16 (1.08–4.32) | 0.03 | | 1.91 (0.94–3.89) | 0.073 | 1.63 (0.74–3.56) | 0.223 | | 1.34 (0.63–2.85) | 0.45 |
| 2^nd^ (2.0 to 6.0) (n = 559) | 3.05 (1.60–5.83) | 0.001 | | 3.32 (1.66–6.22) | 0.001 | 2.68 (1.25–5.72) | 0.011 | | 1.93 (0.90–4.13) | 0.09 |
| 3^rd^ (> 6.0) (n = 565) | 5.38 (2.56–11.30) | < 0.001 | | 5.15 (2.43–10.90) | < 0.001 | 3.85 (1.70–8.71) | 0.001 | | 3.71 (1.64–8.41) | 0.002 |

RR, relative risk; CI, confidence interval; WC, waist circumference.

Model 1: no adjustment.

Model 2: adjusted for age, smoking, and alcohol drinking at baseline.

Model 3: adjusted for the variables in model 2 plus ALT, TG, HDL-C, BMI, and systolic BP at baseline.

Model 4: adjusted for the variables in model 3 plus HbA1c at baseline.

**Supplementary Table 3. RRs of Diabetes According to Variables That Included Adjustment**

| **Variables** | **Diabetes** | |
| --- | --- | --- |
|  | **RR (95% CI)** | ***P*** |
| Age | 1.02 (1.01–1.03) | < 0.001 |
| Sex, female | 0.67 (0.54–0.84) | < 0.001 |
| Baseline BMI | 1.11 (1.09–1.13) | < 0.001 |
| Baseline WC | 1.05 (1.04–1.06) | < 0.001 |
| HbA1c | 39.45 (28.62–54.37) | < 0.001 |
| TG | 1.00(1.00–1.00) | < 0.001 |
| HDL-cholesterol | 0.97(0.94–0.98) | < 0.001 |
| ALT | 1.00(1.00–1.00) | < 0.001 |
| Smoking | 1.46 (1.21–1.76) | < 0.001 |
| Alcohol drinking | 1.02 (0.86–1.22) | 0.812 |
| Systolic BP | 1.01 (1.00–1.01) | 0.109 |

**Supplementary Table 4. RRs of Diabetes According to NAFLD and WC Changes in the Subjects with Over 2 Years Follow-up.**

|  | **Model 1** | | **Model 2** | | **Model 3** | | **Model 4** | |
| --- | --- | --- | --- | --- | --- | --- | --- | --- |
|  | **RR (95% CI)** | ***P*** | **RR (95% CI)** | ***P*** | **RR (95% CI)** | ***P*** | **RR (95% CI)** | ***P*** |
| Total population (n = 5826) | 3.02 (2.46–3.72) | < 0.001 | 2.92 (2.37–3.61) | < 0.001 | 2.51 (2.02–3.13) | < 0.001 | 2.02 (1.62–2.52) | < 0.001 |
| WC change (cm) |  |  |  |  |  |  |  |  |
| 1^st^ (< 0.0) (n = 1970) | 2.76 (1.94–3.95) | < 0.001 | 2.73 (1.91–3.91) | < 0.001 | 2.19 (1.49–3.22) | < 0.001 | 1.71 (1.17–2.51) | 0.006 |
| 2^nd^ (0.0 to 4.0) (n = 1907) | 3.10 (2.19–4.39) | < 0.001 | 3.01 (2.11–4.29) | < 0.001 | 2.35 (1.63–3.41) | < 0.001 | 1.91 (1.31–2.78) | 0.001 |
| 3^rd^ (> 4.0) (n = 1947) | 3.06 (2.10–4.46) | < 0.001 | 3.00 (2.04–4.40) | < 0.001 | 2.68 (1.80–3.99) | < 0.001 | 2.41 (1.61–3.60) | < 0.001 |

RR, relative risk; CI, confidence interval; WC, waist circumference.

Model 1: no adjustment.

Model 2: adjusted for age, sex, smoking, and alcohol drinking at baseline.

Model 3: adjusted for the variables in model 2 plus ALT, TG, HDL-C, BMI, and systolic BP at baseline.

Model 4: adjusted for the variables in model 3 plus HbA1c at baseline.
